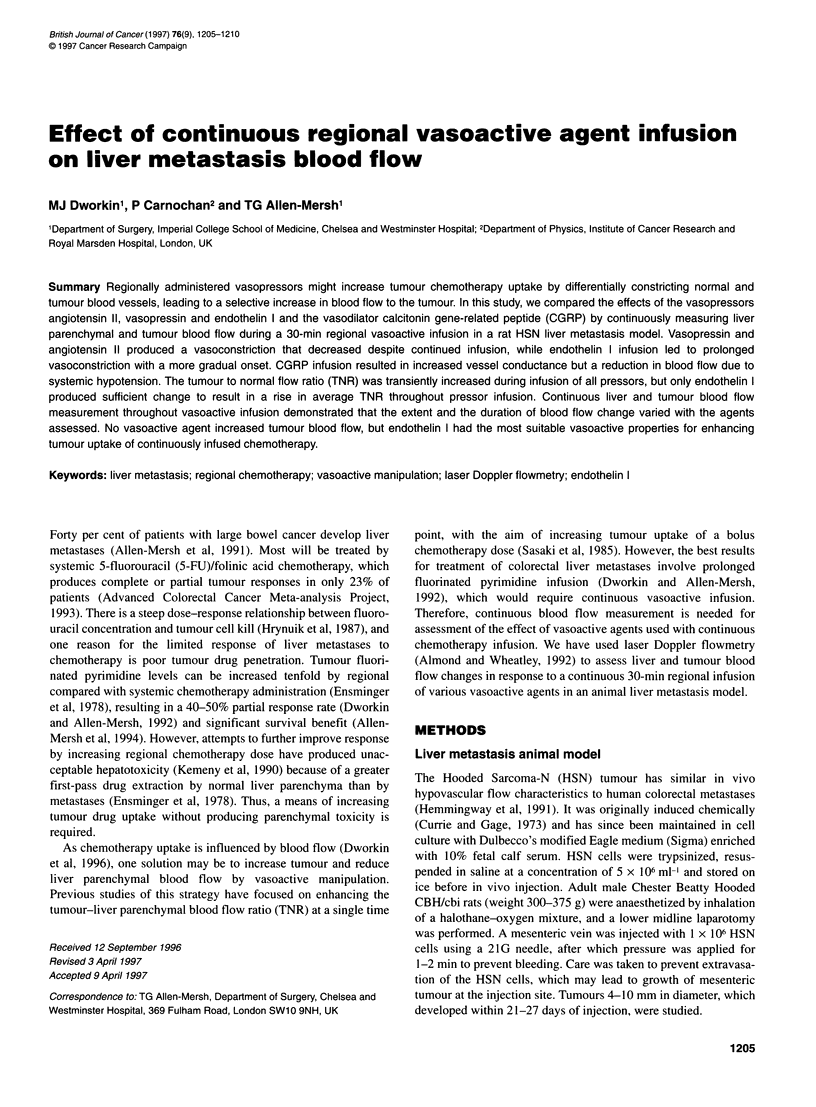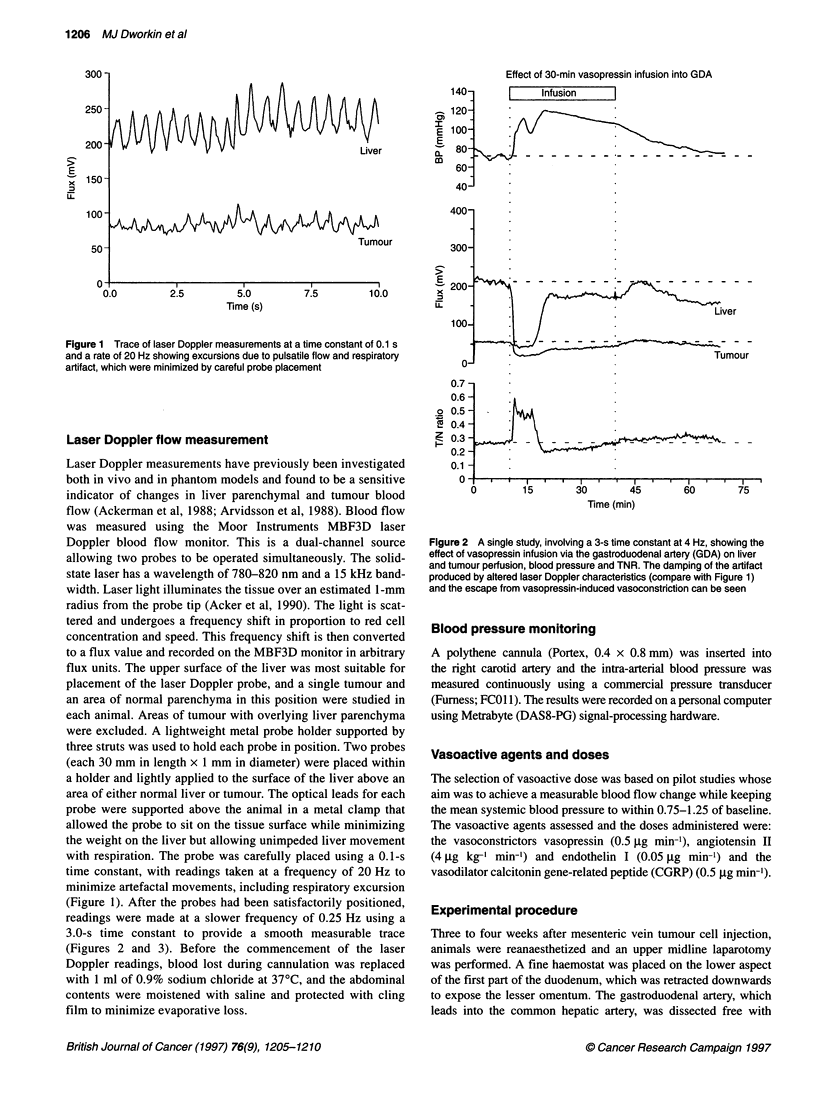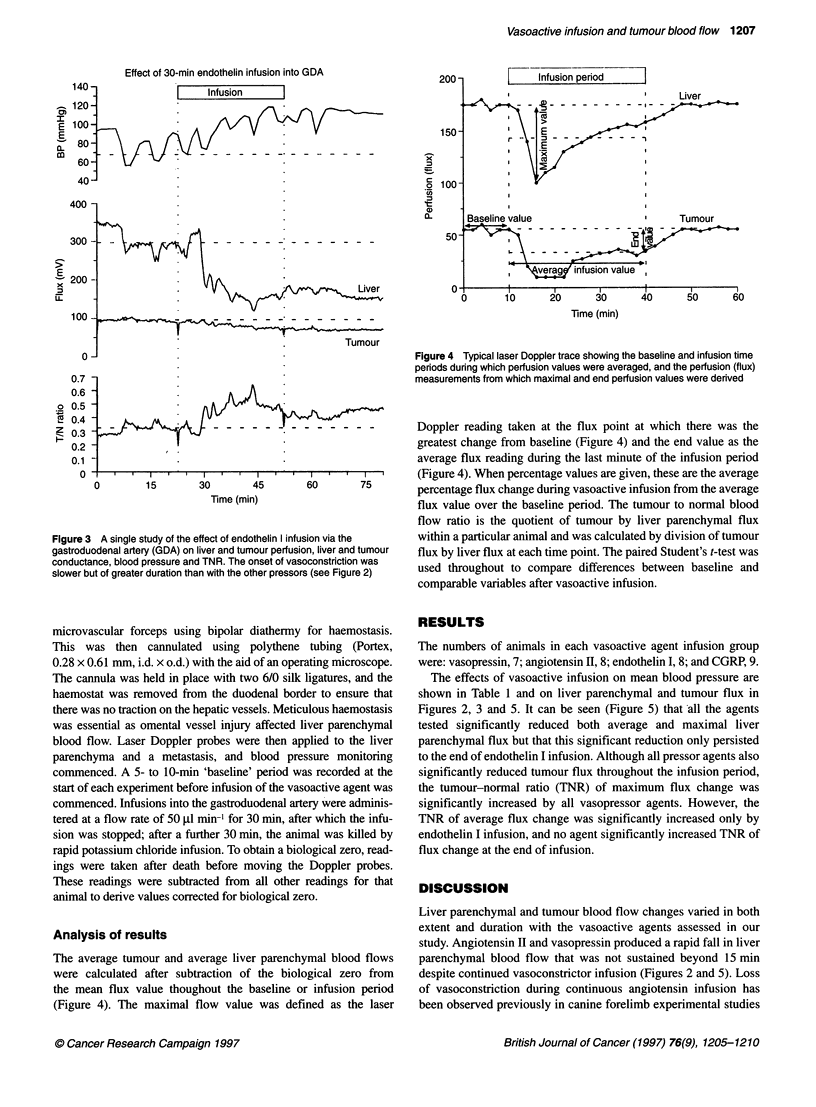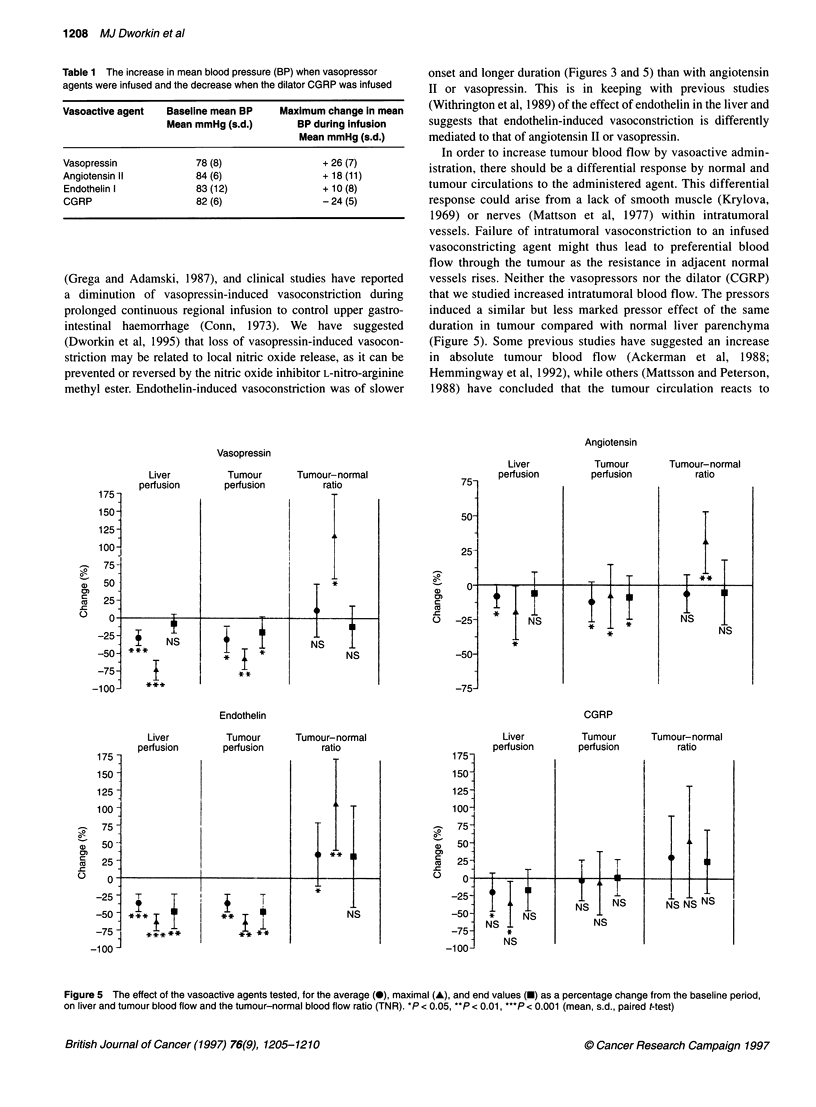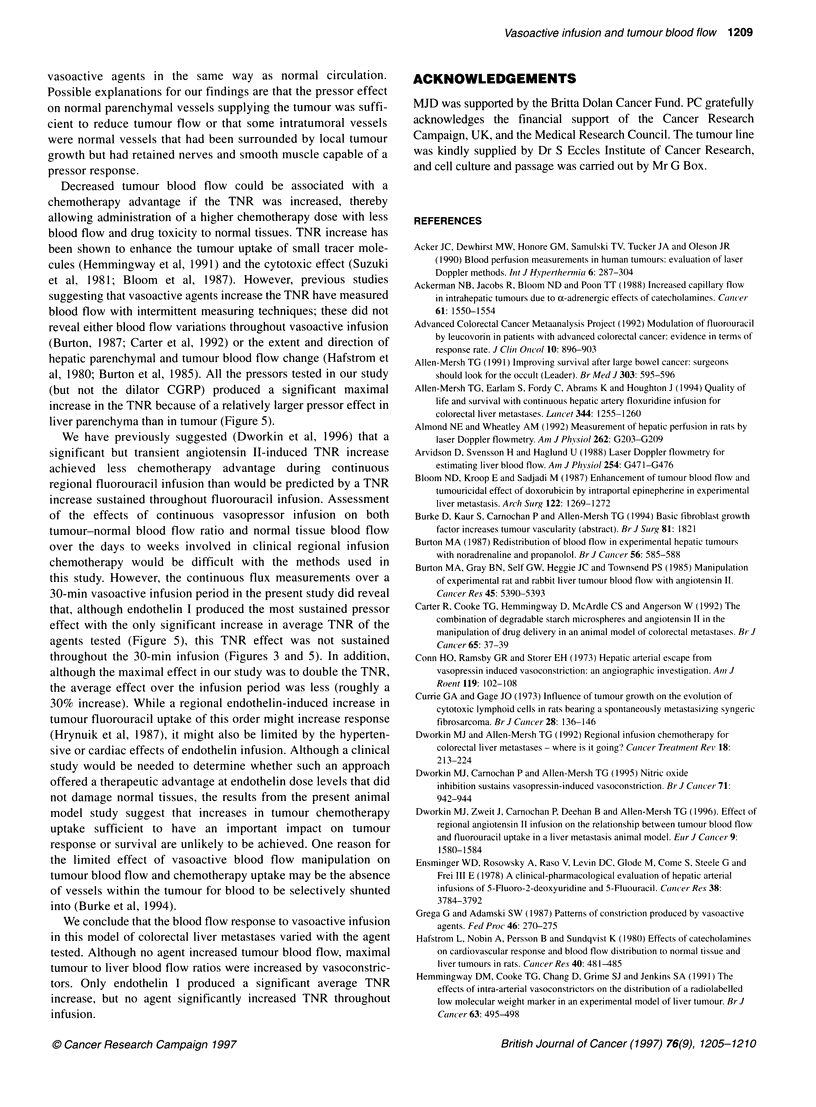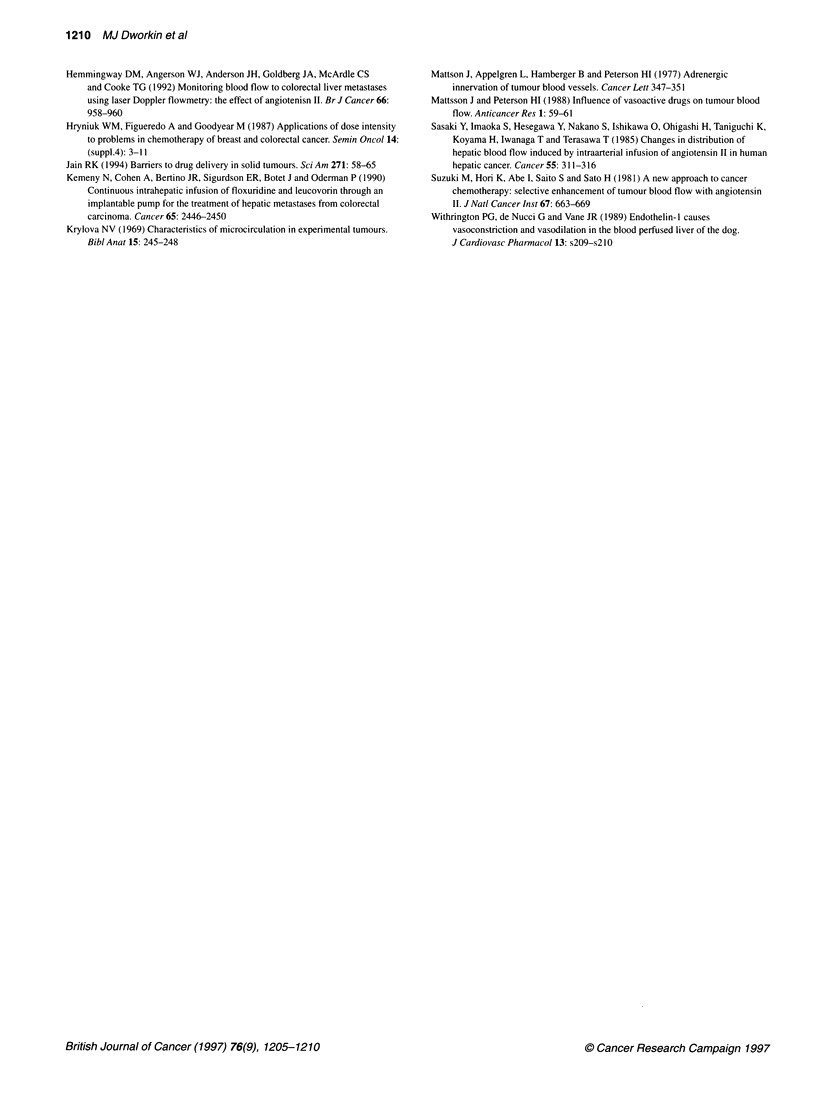# Effect of continuous regional vasoactive agent infusion on liver metastasis blood flow.

**DOI:** 10.1038/bjc.1997.534

**Published:** 1997

**Authors:** M. J. Dworkin, P. Carnochan, T. G. Allen-Mersh

**Affiliations:** Department of Surgery, Imperial College School of Medicine, Chelsea and Westminster Hospital, London, UK.

## Abstract

Regionally administered vasopressors might increase tumour chemotherapy uptake by differentially constricting normal and tumour blood vessels, leading to a selective increase in blood flow to the tumour. In this study, we compared the effects of the vasopressors angiotensin II, vasopressin and endothelin I and the vasodilator calcitonin gene-related peptide (CGRP) by continuously measuring liver parenchymal and tumour blood flow during a 30-min regional vasoactive infusion in a rat HSN liver metastasis model. Vasopressin and angiotensin II produced a vasoconstriction that decreased despite continued infusion, while endothelin I infusion led to prolonged vasoconstriction with a more gradual onset. CGRP infusion resulted in increased vessel conductance but a reduction in blood flow due to systemic hypotension. The tumour to normal flow ratio (TNR) was transiently increased during infusion of all pressors, but only endothelin I produced sufficient change to result in a rise in average TNR throughout pressor infusion. Continuous liver and tumour blood flow measurement throughout vasoactive infusion demonstrated that the extent and the duration of blood flow change varied with the agents assessed. No vasoactive agent increased tumour blood flow, but endothelin I had the most suitable vasoactive properties for enhancing tumour uptake of continuously infused chemotherapy.


British Journal of Cancer (1997) 76(9), 1205–1210
© 1997 Cancer Research Campaign

### Effect of continuous regional vasoactive agent infusion on liver metastasis blood flow

**MJ Dworkin[1], P Carnochan[2] and TG Allen-Mersh[1]**

[1]Department of Surgery, Imperial College School of Medicine, Chelsea and Westminster Hospital; [2]Department of Physics, Institute of Cancer Research and Royal Marsden Hospital, London, UK

**Summary** Regionally administered vasopressors might increase tumour chemotherapy uptake by differentially constricting normal and tumour blood vessels, leading to a selective increase in blood flow to the tumour. In this study, we compared the effects of the vasopressors angiotensin II, vasopressin and endothelin I and the vasodilator calcitonin gene-related peptide (CGRP) by continuously measuring liver parenchymal and tumour blood flow during a 30-min regional vasoactive infusion in a rat HSN liver metastasis model. Vasopressin and angiotensin II produced a vasoconstriction that decreased despite continued infusion, while endothelin I infusion led to prolonged vasoconstriction with a more gradual onset. CGRP infusion resulted in increased vessel conductance but a reduction in blood flow due to systemic hypotension. The tumour to normal flow ratio (TNR) was transiently increased during infusion of all pressors, but only endothelin I produced sufficient change to result in a rise in average TNR throughout pressor infusion. Continuous liver and tumour blood flow measurement throughout vasoactive infusion demonstrated that the extent and the duration of blood flow change varied with the agents assessed. No vasoactive agent increased tumour blood flow, but endothelin I had the most suitable vasoactive properties for enhancing tumour uptake of continuously infused chemotherapy.

**Keywords:** liver metastasis; regional chemotherapy; vasoactive manipulation; laser Doppler flowmetry; endothelin I

Forty per cent of patients with large bowel cancer develop liver metastases (Allen-Mersh et al, 1991). Most will be treated by systemic 5-fluorouracil (5-FU)/folinic acid chemotherapy, which produces complete or partial tumour responses in only 23% of patients (Advanced Colorectal Cancer Meta-analysis Project, 1993). There is a steep dose–response relationship between fluorouracil concentration and tumour cell kill (Hrynuik et al, 1987), and one reason for the limited response of liver metastases to chemotherapy is poor tumour drug penetration. Tumour fluorinated pyrimidine levels can be increased tenfold by regional compared with systemic chemotherapy administration (Ensminger et al, 1978), resulting in a 40–50% partial response rate (Dworkin and Allen-Mersh, 1992) and significant survival benefit (Allen-Mersh et al, 1994). However, attempts to further improve response by increasing regional chemotherapy dose have produced unacceptable hepatotoxicity (Kemeny et al, 1990) because of a greater first-pass drug extraction by normal liver parenchyma than by metastases (Ensminger et al, 1978). Thus, a means of increasing tumour drug uptake without producing parenchymal toxicity is required.

As chemotherapy uptake is influenced by blood flow (Dworkin et al, 1996), one solution may be to increase tumour and reduce liver parenchymal blood flow by vasoactive manipulation. Previous studies of this strategy have focused on enhancing the tumour–liver parenchymal blood flow ratio (TNR) at a single time point, with the aim of increasing tumour uptake of a bolus chemotherapy dose (Sasaki et al, 1985). However, the best results for treatment of colorectal liver metastases involve prolonged fluorinated pyrimidine infusion (Dworkin and Allen-Mersh, 1992), which would require continuous vasoactive infusion. Therefore, continuous blood flow measurement is needed for assessment of the effect of vasoactive agents used with continuous chemotherapy infusion. We have used laser Doppler flowmetry (Almond and Wheatley, 1992) to assess liver and tumour blood flow changes in response to a continuous 30-min regional infusion of various vasoactive agents in an animal liver metastasis model.

*Received 12 September 1996*
*Revised 3 April 1997*
*Accepted 9 April 1997*

*Correspondence to:* TG Allen-Mersh, Department of Surgery, Chelsea and Westminster Hospital, 369 Fulham Road, London SW10 9NH, UK

### METHODS

### Liver metastasis animal model

The Hooded Sarcoma-N (HSN) tumour has similar in vivo hypovascular flow characteristics to human colorectal metastases (Hemmingway et al, 1991). It was originally induced chemically (Currie and Gage, 1973) and has since been maintained in cell culture with Dulbecco's modified Eagle medium (Sigma) enriched with 10% fetal calf serum. HSN cells were trypsinized, resuspended in saline at a concentration of $5 \times 10^6$ ml$^{-1}$ and stored on ice before in vivo injection. Adult male Chester Beatty Hooded CBH/cbi rats (weight 300–375 g) were anaesthetized by inhalation of a halothane–oxygen mixture, and a lower midline laparotomy was performed. A mesenteric vein was injected with $1 \times 10^6$ HSN cells using a 21G needle, after which pressure was applied for 1–2 min to prevent bleeding. Care was taken to prevent extravasation of the HSN cells, which may lead to growth of mesenteric tumour at the injection site. Tumours 4–10 mm in diameter, which developed within 21–27 days of injection, were studied.

**Figure 1** Trace of laser Doppler measurements at a time constant of 0.1 s and a rate of 20 Hz showing excursions due to pulsatile flow and respiratory artifact, which were minimized by careful probe placement

### Laser Doppler flow measurement

Laser Doppler measurements have previously been investigated both in vivo and in phantom models and found to be a sensitive indicator of changes in liver parenchymal and tumour blood flow (Ackerman et al, 1988; Arvidsson et al, 1988). Blood flow was measured using the Moor Instruments MBF3D laser Doppler blood flow monitor. This is a dual-channel source allowing two probes to be operated simultaneously. The solid-state laser has a wavelength of 780–820 nm and a 15 kHz bandwidth. Laser light illuminates the tissue over an estimated 1-mm radius from the probe tip (Acker et al, 1990). The light is scattered and undergoes a frequency shift in proportion to red cell concentration and speed. This frequency shift is then converted to a flux value and recorded on the MBF3D monitor in arbitrary flux units. The upper surface of the liver was most suitable for placement of the laser Doppler probe, and a single tumour and an area of normal parenchyma in this position were studied in each animal. Areas of tumour with overlying liver parenchyma were excluded. A lightweight metal probe holder supported by three struts was used to hold each probe in position. Two probes (each 30 mm in length × 1 mm in diameter) were placed within a holder and lightly applied to the surface of the liver above an area of either normal liver or tumour. The optical leads for each probe were supported above the animal in a metal clamp that allowed the probe to sit on the tissue surface while minimizing the weight on the liver but allowing unimpeded liver movement with respiration. The probe was carefully placed using a 0.1-s time constant, with readings taken at a frequency of 20 Hz to minimize artefactal movements, including respiratory excursion (Figure 1). After the probes had been satisfactorily positioned, readings were made at a slower frequency of 0.25 Hz using a 3.0-s time constant to provide a smooth measurable trace (Figures 2 and 3). Before the commencement of the laser Doppler readings, blood lost during cannulation was replaced with 1 ml of 0.9% sodium chloride at 37°C, and the abdominal contents were moistened with saline and protected with cling film to minimize evaporative loss.

**Figure 2** A single study, involving a 3-s time constant at 4 Hz, showing the effect of vasopressin infusion via the gastroduodenal artery (GDA) on liver and tumour perfusion, blood pressure and TNR. The damping of the artifact produced by altered laser Doppler characteristics (compare with Figure 1) and the escape from vasopressin-induced vasoconstriction can be seen

### Blood pressure monitoring

A polythene cannula (Portex, 0.4 × 0.8 mm) was inserted into the right carotid artery and the intra-arterial blood pressure was measured continuously using a commercial pressure transducer (Furness; FC011). The results were recorded on a personal computer using Metrabyte (DAS8-PG) signal-processing hardware.

### Vasoactive agents and doses

The selection of vasoactive dose was based on pilot studies whose aim was to achieve a measurable blood flow change while keeping the mean systemic blood pressure to within 0.75–1.25 of baseline. The vasoactive agents assessed and the doses administered were: the vasoconstrictors vasopressin (0.5 $\mu$g min$^{-1}$), angiotensin II (4 $\mu$g kg$^{-1}$ min$^{-1}$) and endothelin I (0.05 $\mu$g min$^{-1}$) and the vasodilator calcitonin gene-related peptide (CGRP) (0.5 $\mu$g min$^{-1}$).

### Experimental procedure

Three to four weeks after mesenteric vein tumour cell injection, animals were reanaesthetized and an upper midline laparotomy was performed. A fine haemostat was placed on the lower aspect of the first part of the duodenum, which was retracted downwards to expose the lesser omentum. The gastroduodenal artery, which leads into the common hepatic artery, was dissected free with

© Cancer Research Campaign 1997

**Figure 3** A single study of the effect of endothelin I infusion via the gastroduodenal artery (GDA) on liver and tumour perfusion, liver and tumour conductance, blood pressure and TNR. The onset of vasoconstriction was slower but of greater duration than with the other pressors (see Figure 2)

**Figure 4** Typical laser Doppler trace showing the baseline and infusion time periods during which perfusion values were averaged, and the perfusion (flux) measurements from which maximal and end perfusion values were derived microvascular forceps using bipolar diathermy for haemostasis. This was then cannulated using polythene tubing (Portex, 0.28 × 0.61 mm, i.d. × o.d.) with the aid of an operating microscope. The cannula was held in place with two 6/0 silk ligatures, and the haemostat was removed from the duodenal border to ensure that there was no traction on the hepatic vessels. Meticulous haemostasis was essential as omental vessel injury affected liver parenchymal blood flow. Laser Doppler probes were then applied to the liver parenchyma and a metastasis, and blood pressure monitoring commenced. A 5- to 10-min 'baseline' period was recorded at the start of each experiment before infusion of the vasoactive agent was commenced. Infusions into the gastroduodenal artery were administered at a flow rate of 50 μl $min^{-1}$ for 30 min, after which the infusion was stopped; after a further 30 min, the animal was killed by rapid potassium chloride infusion. To obtain a biological zero, readings were taken after death before moving the Doppler probes. These readings were subtracted from all other readings for that animal to derive values corrected for biological zero.

### Analysis of results

The average tumour and average liver parenchymal blood flows were calculated after subtraction of the biological zero from the mean flux value thoughout the baseline or infusion period (Figure 4). The maximal flow value was defined as the laser Doppler reading taken at the flux point at which there was the greatest change from baseline (Figure 4) and the end value as the average flux reading during the last minute of the infusion period (Figure 4). When percentage values are given, these are the average percentage flux change during vasoactive infusion from the average flux value over the baseline period. The tumour to normal blood flow ratio is the quotient of tumour by liver parenchymal flux within a particular animal and was calculated by division of tumour flux by liver flux at each time point. The paired Student's *t*-test was used throughout to compare differences between baseline and comparable variables after vasoactive infusion.

### RESULTS

The numbers of animals in each vasoactive agent infusion group were: vasopressin, 7; angiotensin II, 8; endothelin I, 8; and CGRP, 9.

The effects of vasoactive infusion on mean blood pressure are shown in Table 1 and on liver parenchymal and tumour flux in Figures 2, 3 and 5. It can be seen (Figure 5) that all the agents tested significantly reduced both average and maximal liver parenchymal flux but that this significant reduction only persisted to the end of endothelin I infusion. Although all pressor agents also significantly reduced tumour flux throughout the infusion period, the tumour–normal ratio (TNR) of maximum flux change was significantly increased by all vasopressor agents. However, the TNR of average flux change was significantly increased only by endothelin I infusion, and no agent significantly increased TNR of flux change at the end of infusion.

### DISCUSSION

Liver parenchymal and tumour blood flow changes varied in both extent and duration with the vasoactive agents assessed in our study. Angiotensin II and vasopressin produced a rapid fall in liver parenchymal blood flow that was not sustained beyond 15 min despite continued vasoconstrictor infusion (Figures 2 and 5). Loss of vasoconstriction during continuous angiotensin infusion has been observed previously in canine forelimb experimental studies

© Cancer Research Campaign 1997

**Table 1** The increase in mean blood pressure (BP) when vasopressor agents were infused and the decrease when the dilator CGRP was infused

| Vasoactive agent | Baseline mean BP Mean mmHg (s.d.) | Maximum change in mean BP during infusion Mean mmHg (s.d.) |
|---|---|---|
| Vasopressin | 78 (8) | + 26 (7) |
| Angiotensin II | 84 (6) | + 18 (11) |
| Endothelin I | 83 (12) | + 10 (8) |
| CGRP | 82 (6) | – 24 (5) |

(Grega and Adamski, 1987), and clinical studies have reported a diminution of vasopressin-induced vasoconstriction during prolonged continuous regional infusion to control upper gastro-intestinal haemorrhage (Conn, 1973). We have suggested (Dworkin et al, 1995) that loss of vasopressin-induced vasoconstriction may be related to local nitric oxide release, as it can be prevented or reversed by the nitric oxide inhibitor L-nitro-arginine methyl ester. Endothelin-induced vasoconstriction was of slower onset and longer duration (Figures 3 and 5) than with angiotensin II or vasopressin. This is in keeping with previous studies (Withrington et al, 1989) of the effect of endothelin in the liver and suggests that endothelin-induced vasoconstriction is differently mediated to that of angiotensin II or vasopressin.

In order to increase tumour blood flow by vasoactive administration, there should be a differential response by normal and tumour circulations to the administered agent. This differential response could arise from a lack of smooth muscle (Krylova, 1969) or nerves (Mattson et al, 1977) within intratumoral vessels. Failure of intratumoral vasoconstriction to an infused vasoconstricting agent might thus lead to preferential blood flow through the tumour as the resistance in adjacent normal vessels rises. Neither the vasopressors nor the dilator (CGRP) that we studied increased intratumoral blood flow. The pressors induced a similar but less marked pressor effect of the same duration in tumour compared with normal liver parenchyma (Figure 5). Some previous studies have suggested an increase in absolute tumour blood flow (Ackerman et al, 1988; Hemmingway et al, 1992), while others (Mattsson and Peterson, 1988) have concluded that the tumour circulation reacts to

**Figure 5** The effect of the vasoactive agents tested, for the average (●), maximal (▲), and end values (■) as a percentage change from the baseline period, on liver and tumour blood flow and the tumour–normal blood flow ratio (TNR). *$P < 0.05$, **$P < 0.01$, ***$P < 0.001$ (mean, s.d., paired *t*-test)

© Cancer Research Campaign 1997

vasoactive agents in the same way as normal circulation. Possible explanations for our findings are that the pressor effect on normal parenchymal vessels supplying the tumour was sufficient to reduce tumour flow or that some intratumoral vessels were normal vessels that had been surrounded by local tumour growth but had retained nerves and smooth muscle capable of a pressor response.

Decreased tumour blood flow could be associated with a chemotherapy advantage if the TNR was increased, thereby allowing administration of a higher chemotherapy dose with less blood flow and drug toxicity to normal tissues. TNR increase has been shown to enhance the tumour uptake of small tracer molecules (Hemmingway et al, 1991) and the cytotoxic effect (Suzuki et al, 1981; Bloom et al, 1987). However, previous studies suggesting that vasoactive agents increase the TNR have measured blood flow with intermittent measuring techniques; these did not reveal either blood flow variations throughout vasoactive infusion (Burton, 1987; Carter et al, 1992) or the extent and direction of hepatic parenchymal and tumour blood flow change (Hafstrom et al, 1980; Burton et al, 1985). All the pressors tested in our study (but not the dilator CGRP) produced a significant maximal increase in the TNR because of a relatively larger pressor effect in liver parenchyma than in tumour (Figure 5).

We have previously suggested (Dworkin et al, 1996) that a significant but transient angiotensin II-induced TNR increase achieved less chemotherapy advantage during continuous regional fluorouracil infusion than would be predicted by a TNR increase sustained throughout fluorouracil infusion. Assessment of the effects of continuous vasopressor infusion on both tumour–normal blood flow ratio and normal tissue blood flow over the days to weeks involved in clinical regional infusion chemotherapy would be difficult with the methods used in this study. However, the continuous flux measurements over a 30-min vasoactive infusion period in the present study did reveal that, although endothelin I produced the most sustained pressor effect with the only significant increase in average TNR of the agents tested (Figure 5), this TNR effect was not sustained throughout the 30-min infusion (Figures 3 and 5). In addition, although the maximal effect in our study was to double the TNR, the average effect over the infusion period was less (roughly a 30% increase). While a regional endothelin-induced increase in tumour fluorouracil uptake of this order might increase response (Hrynuik et al, 1987), it might also be limited by the hypertensive or cardiac effects of endothelin infusion. Although a clinical study would be needed to determine whether such an approach offered a therapeutic advantage at endothelin dose levels that did not damage normal tissues, the results from the present animal model study suggest that increases in tumour chemotherapy uptake sufficient to have an important impact on tumour response or survival are unlikely to be achieved. One reason for the limited effect of vasoactive blood flow manipulation on tumour blood flow and chemotherapy uptake may be the absence of vessels within the tumour for blood to be selectively shunted into (Burke et al, 1994).

We conclude that the blood flow response to vasoactive infusion in this model of colorectal liver metastases varied with the agent tested. Although no agent increased tumour blood flow, maximal tumour to liver blood flow ratios were increased by vasoconstrictors. Only endothelin I produced a significant average TNR increase, but no agent significantly increased TNR throughout infusion.

### ACKNOWLEDGEMENTS

MJD was supported by the Britta Dolan Cancer Fund. PC gratefully acknowledges the financial support of the Cancer Research Campaign, UK, and the Medical Research Council. The tumour line was kindly supplied by Dr S Eccles Institute of Cancer Research, and cell culture and passage was carried out by Mr G Box.

### REFERENCES

Acker JC, Dewhirst MW, Honore GM, Samulski TV, Tucker JA and Oleson JR (1990) Blood perfusion measurements in human tumours: evaluation of laser Doppler methods. *Int J Hyperthermia* **6**: 287–304

Ackerman NB, Jacobs R, Bloom ND and Poon TT (1988) Increased capillary flow in intrahepatic tumours due to α-adrenergic effects of catecholamines. *Cancer* **61**: 1550–1554

Advanced Colorectal Cancer Metaanalysis Project (1992) Modulation of fluorouracil by leucovorin in patients with advanced colorectal cancer: evidence in terms of response rate. *J Clin Oncol* **10**: 896–903

Allen-Mersh TG (1991) Improving survival after large bowel cancer: surgeons should look for the occult (Leader). *Br Med J* **303**: 595–596

Allen-Mersh TG, Earlam S, Fordy C, Abrams K and Houghton J (1994) Quality of life and survival with continuous hepatic artery floxuridine infusion for colorectal liver metastases. *Lancet* **344**: 1255–1260

Almond NE and Wheatley AM (1992) Measurement of hepatic perfusion in rats by laser Doppler flowmetry. *Am J Physiol* **262**: G203–G209

Arvidson D, Svensson H and Haglund U (1988) Laser Doppler flowmetry for estimating liver blood flow. *Am J Physiol* **254**: G471–G476

Bloom ND, Kroop E and Sadjadi M (1987) Enhancement of tumour blood flow and tumouricidal effect of doxorubicin by intraportal epinepherine in experimental liver metastasis. *Arch Surg* **122**: 1269–1272

Burke D, Kaur S, Carnochan P and Allen-Mersh TG (1994) Basic fibroblast growth factor increases tumour vascularity (abstract). *Br J Surg* **81**: 1821

Burton MA (1987) Redistribution of blood flow in experimental hepatic tumours with noradrenaline and propanolol. *Br J Cancer* **56**: 585–588

Burton MA, Gray BN, Self GW, Heggie JC and Townsend PS (1985) Manipulation of experimental rat and rabbit liver tumour blood flow with angiotensin II. *Cancer Res* **45**: 5390–5393

Carter R, Cooke TG, Hemmingway D, McArdle CS and Angerson W (1992) The combination of degradable starch microspheres and angiotensin II in the manipulation of drug delivery in an animal model of colorectal metastases. *Br J Cancer* **65**: 37–39

Conn HO, Ramsby GR and Storer EH (1973) Hepatic arterial escape from vasopressin induced vasoconstriction: an angiographic investigation. *Am J Roent* **119**: 102–108

Currie GA and Gage JO (1973) Influence of tumour growth on the evolution of cytotoxic lymphoid cells in rats bearing a spontaneously metastasizing syngeric fibrosarcoma. *Br J Cancer* **28**: 136–146

Dworkin MJ and Allen-Mersh TG (1992) Regional infusion chemotherapy for colorectal liver metastases – where is it going? *Cancer Treatment Rev* **18**: 213–224

Dworkin MJ, Carnochan P and Allen-Mersh TG (1995) Nitric oxide inhibition sustains vasopressin-induced vasoconstriction. *Br J Cancer* **71**: 942–944

Dworkin MJ, Zweit J, Carnochan P, Deehan B and Allen-Mersh TG (1996). Effect of regional angiotensin II infusion on the relationship between tumour blood flow and fluorouracil uptake in a liver metastasis animal model. *Eur J Cancer* **9**: 1580–1584

Ensminger WD, Rosowsky A, Raso V, Levin DC, Glode M, Come S, Steele G and Frei III E (1978) A clinical-pharmacological evaluation of hepatic arterial infusions of 5-Fluoro-2-deoxyuridine and 5-Fluouracil. *Cancer Res* **38**: 3784–3792

Grega G and Adamski SW (1987) Patterns of constriction produced by vasoactive agents. *Fed Proc* **46**: 270–275

Hafstrom L, Nobin A, Persson B and Sundqvist K (1980) Effects of catecholamines on cardiovascular response and blood flow distribution to normal tissue and liver tumours in rats. *Cancer Res* **40**: 481–485

Hemmingway DM, Cooke TG, Chang D, Grime SJ and Jenkins SA (1991) The effects of intra-arterial vasoconstrictors on the distribution of a radiolabelled low molecular weight marker in an experimental model of liver tumour. *Br J Cancer* **63**: 495–498

© Cancer Research Campaign 1997

Hemmingway DM, Angerson WJ, Anderson JH, Goldberg JA, McArdle CS and Cooke TG (1992) Monitoring blood flow to colorectal liver metastases using laser Doppler flowmetry: the effect of angiotenisn II. *Br J Cancer* **66**: 958–960

Hryniuk WM, Figueredo A and Goodyear M (1987) Applications of dose intensity to problems in chemotherapy of breast and colorectal cancer. *Semin Oncol* **14**: (suppl.4): 3–11

Jain RK (1994) Barriers to drug delivery in solid tumours. *Sci Am* **271**: 58–65

Kemeny N, Cohen A, Bertino JR, Sigurdson ER, Botet J and Oderman P (1990) Continuous intrahepatic infusion of floxuridine and leucovorin through an implantable pump for the treatment of hepatic metastases from colorectal carcinoma. *Cancer* **65**: 2446–2450

Krylova NV (1969) Characteristics of microcirculation in experimental tumours. *Bibl Anat* **15**: 245–248

Mattson J, Appelgren L, Hamberger B and Peterson HI (1977) Adrenergic innervation of tumour blood vessels. *Cancer Lett* 347–351

Mattsson J and Peterson HI (1988) Influence of vasoactive drugs on tumour blood flow. *Anticancer Res* **1**: 59–61

Sasaki Y, Imaoka S, Hesegawa Y, Nakano S, Ishikawa O, Ohigashi H, Taniguchi K, Koyama H, Iwanaga T and Terasawa T (1985) Changes in distribution of hepatic blood flow induced by intraarterial infusion of angiotensin II in human hepatic cancer. *Cancer* **55**: 311–316

Suzuki M, Hori K, Abe I, Saito S and Sato H (1981) A new approach to cancer chemotherapy: selective enhancement of tumour blood flow with angiotensin II. *J Natl Cancer Inst* **67**: 663–669

Withrington PG, de Nucci G and Vane JR (1989) Endothelin-1 causes vasoconstriction and vasodilation in the blood perfused liver of the dog. *J Cardiovasc Pharmacol* **13**: s209–s210

*© Cancer Research Campaign 1997*